# *Acinetobacter baumannii*: An Ancient Commensal with Weapons of a Pathogen

**DOI:** 10.3390/pathogens10040387

**Published:** 2021-03-24

**Authors:** Meysam Sarshar, Payam Behzadi, Daniela Scribano, Anna Teresa Palamara, Cecilia Ambrosi

**Affiliations:** 1 Research Laboratories, Bambino Gesù Children’s Hospital, IRCCS, 00146 Rome, Italy; meysam.sarshar@uniroma1.it; 2 Department of Microbiology, College of Basic Sciences, Shahr-e-Qods Branch, Islamic Azad University, Tehran 37541-374, Iran; behzadipayam@yahoo.com; 3 Department of Public Health and Infectious Diseases, Sapienza University of Rome, 00185 Rome, Italy; 4 Dani Di Giò Foundation-Onlus, 00193 Rome, Italy; 5 Laboratory affiliated to Institute Pasteur Italia- Cenci Bolognetti Foundation, Department of Public Health and Infectious Diseases, Sapienza University of Rome, 00185 Rome, Italy; annateresa.palamara@uniroma1.it; 6 IRCCS San Raffaele Pisana, 00166 Rome, Italy; 7 Department of Human Sciences and Promotion of the Quality of Life, San Raffaele Open University, 00166 Rome, Italy

**Keywords:** *Acinetobacter baumannii*, community-acquired infections, nosocomial infections, adherence, persistence, virulence factors, invasion, internalization

## Abstract

*Acinetobacter baumannii* is regarded as a life-threatening pathogen associated with community-acquired and nosocomial infections, mainly pneumonia. The rise in the number of *A. baumannii* antibiotic-resistant strains reduces effective therapies and increases mortality. Bacterial comparative genomic studies have unraveled the innate and acquired virulence factors of *A. baumannii.* These virulence factors are involved in antibiotic resistance, environmental persistence, host-pathogen interactions, and immune evasion. Studies on host–pathogen interactions revealed that *A. baumannii* evolved different mechanisms to adhere to in order to invade host respiratory cells as well as evade the host immune system. In this review, we discuss current data on *A. baumannii* genetic features and virulence factors. An emphasis is given to the players in host–pathogen interaction in the respiratory tract. In addition, we report recent investigations into host defense systems using in vitro and in vivo models, providing new insights into the innate immune response to *A. baumannii* infections. Increasing our knowledge of *A. baumannii* pathogenesis may help the development of novel therapeutic strategies based on anti-adhesive, anti-virulence, and anti-cell to cell signaling pathways drugs.

## 1. Introduction

The studies on *Acinetobacter* spp. began in 1911 when it was isolated from a soil sample and named *Micrococcus calcoaceticus* (Henriksen [1]). Only in 1971 was the genus *Acinetobacter* officially recognized by taxonomists based on shared biochemical features [1,2,3]. The name of this genus comes from the Greek word a-kinetos-bacter which means non-motile rod, although they do exhibit a coccobacillary morphology and twitching motility. To date, molecular approaches have allowed identification of over 65 validly published species within the *Acinetobacter* genus (https://lpsn.dsmz.de/genus/Acinetobacter; up to 18 March 2021). These bacteria are Gram-negative, strictly aerobic, non-fermentative, oxidase-negative, catalase-positive and non-pigmented or pale yellow to gray pigmented [4,5,6]. Closely related species displaying similar phenotypic and biochemical properties are included in the *Acinetobacter calcoaceticus*–*Acinetobacter baumannii* complex (ACB complex), including *Acinetobacter calcoaceticus*, *Acinetobacter baumannii*, *Acinetobacter pittii*, *Acinetobacter nosocomialis, Acinetobacter seifertii* and *Acinetobacter dijkshoorniae* for which molecular methods of identification are required [7,8,9]. Apart from *A. calcoaceticus*, the other five species are associated with human diseases, with *A. baumannii* as the commonest clinical species around the world [10]. Indeed, this opportunistic pathogen causes community and nosocomial infections, predominantly ventilator-associated pneumonia and bloodstream, urinary tract and skin and soft tissue infections, especially among critically ill patients in intensive care units (ICUs) [11]. Regrettably, the number of multidrug-resistant (MDR) *A. baumannii* isolates has increased significantly [12,13,14]. Apart from innate resistance to several antibiotics, *A. baumannii* genomic plasticity is suited for acquiring or upregulating resistance genes, thereby curtailing effective therapeutic options and increasing mortality rates [14,15]. In addition to antibiotic-resistance genes, *A. baumannii* has several potential virulence traits that allow this bacterium to persist in the environment, adhere to biotic surfaces, invade host cells and escape from the human host immune system [11,16,17,18,19]. While several reviews have described the mechanisms of antibiotic resistance and ability to form biofilms on abiotic surfaces, fewer studies have focused on host–*A. baumannii* interactions. Therefore, this review summarizes the current knowledge of *A. baumannii* genomic features and key bacterial factors that enhance its virulence and mediate its interaction with host cells. We will also disclose recent investigations on how these virulence factors help *A. baumannii* to escape from the human host immune system. 

## 2. *A. baumannii* Pan-Genomics, Genomics and Genome Plasticity

As the number of *A. baumannii* infections increased, it was immediately clear that isolates displayed a remarkable difference in antibiotic profile. Therefore, several investigations were focused on genotypic and phenotypic characterizations of *A. baumannii* isolates due to the global high rate of infections and the paucity of therapeutic options for MDR strains. Two multilocus sequence typing (MLST) schemes were introduced to study the relationships among *A. baumannii* isolates [20,21] (https://pubmlst.org/organisms/Acinetobacter-baumannii) (accessed on 18 March 2021). These analyses highlighted the distribution and spread of different bacterial lineages. MLST showed a specific geographical distribution for each of the different lineages as a consequence of *A. baumannii* isolates that underwent local expansions. Currently, among the nine international clones (ICs) identified, IC-I and -II predominate in terms of outbreaks across continents [22,23,24,25]. However, despite its advantages, MLST has provided limited information to infer the genetic relationship among the increasing number of *A. baumannii* isolates [15,26]. With new sequencing technologies, the study of the whole genomes has improved significantly. This approach provided the opportunity to understand the extent of the genomic heterogeneity among *A. baumannii* isolates, in terms of insertions, deletions, inversions and single nucleotide polymorphisms [22,23,24]. The considerable genetic differences reflect *A. baumannii’s* high genome plasticity that allows acquisition of exogenous genetic information, mostly via horizontal gene transfer and allelic recombination at specific hotspots, along with the loss of unnecessary genes [24,27,28,29]. These data corroborate the knowledge that *A. baumannii* isolates display a high degree of gene diversity to better adapt to new niches; the bacterial environmental or pathogenic lifestyle drives genetic differences, considering environmental strains those mainly isolated in hospitals (i.e., inert surfaces) and pathogenic strains those associated with the human host. To gain insights into *A. baumannii* adaptation to specific niches, most studies have focused on its pangenome. Up to March 2021, a total of 5076 *A. baumannii* genome sequences are available in the NCBI database (www.ncbi.nlm.nih.gov/) (accessed on 18 March 2021). Pangenome encompasses both core and accessory genomes; the core defines the genes commonly shared by all strains included in the analysis, whereas the accessory includes genes retrieved in one (unique gene families) or some strains. A recent work estimated that *A. baumannii* core genome consists of around 2200 genes, whereas the pangenome is notably larger harboring over 19,000 different genes [30]. By analyzing the shared set of genes among 2467 genomes, Mangas et al. distinguished two main *A. baumannii* groups; the first group, accounting for 34% of investigated *A. baumannii* strains, rarely carries plasmids and is characterized by clustered regularly interspaced short palindromic repeat (CRISPR), CRISPR-associated (*cas*) or restriction-modification system (*rms*) genes, and prokaryotic toxin-antitoxin systems (TASs) [30]. TASs are important virulence factors in stressful environmental conditions since they are involved in cellular metabolism, growth activities such as cell cycle process and apoptosis [31,32,33]. Conversely, the second group, accounting for the majority of investigated *A. baumannii* strains (66%) shared a higher number of genes, annotated as plasmid genes with exonuclease activity whereas a limited content of genes involved in biofilm formation was found [30]. It was recently suggested that the presence of CRISPR/Cas systems reduces the acquisition rate of antibiotic resistance genes, being negatively associated with *A. baumannii* MDR isolates [34]. Hence, it can be hypothesized that environmental strains are characterized by the presence of CRISPR/Cas systems that positively influence biofilm production to enhance persistence and inhibit the acquisition of foreign DNA, including antibiotic resistance genes. Vice versa, human-associated strains lose the CRISPR/Cas systems to evolve more easily into MDR strains and reside in antibiotic rich niches. Moreover, if iron acquisition systems are considered, isolates collected from infected samples show a set of genes required for the biosynthesis of acinetobactin, whereas the same set of genes is missing from environmental samples which, in turn, have genes involved in the uptake of iron through xeno-siderophores [35].

Overall, the more that data are acquired from genome sequencing of *A. baumannii* strains, the higher the heterogeneity found among them as a consequence of their high genome plasticity. However, this approach highlighted the genomic divergence of environmental and human-associated strains; although genome sequencing data are biased by the larger number of clinical isolates, it seems that unique strain-specific gene pool arises from genetic changes driven by niche variations [9,21,23,24,27]. Further genome comparison analyses between nonclinical and clinical isolates will help our understanding about the evolutionary process of *A. baumannii* as well as antibiotic-resistance spread and nosocomial persistence. 

## 3. *A. baumannii* and the Treasure of its Virulome

Despite being an opportunistic pathogen, the *A. baumannii* mortality rate of patients with hospital- and community-acquired infections is 23–68% and up to 64%, respectively [11]. Being a major cause of infections, *A. baumannii* belongs to the ESKAPE group together with *Enterococcus faecium*, *Staphylococcus aureus*, *Klebsiella pneumoniae, Pseudomonas aeruginosa* and *Enterobacter* species [36]. The high mortality rate is tightly associated with the worldwide rise in the number of carbapenem-colistin-resistant *A. baumannii* strains [11]. Due to the paucity of therapeutic options in the treatment of *A. baumannii* infections, in 2017 the World Health Organization (WHO) placed this microorganism in the critical priority list of bacteria that urgently require the development of effective drugs as well as alternative strategies (www.who.int) (accessed on 18 March 2021). Since recent papers provide detailed coverage of the genes and mechanisms used by *A. baumannii* to gain resistance to antibiotics, including carbapenems and colistin, this topic will not be discussed in detail and the reader is referred to [37,38,39]. To improve our knowledge of *A. baumannii* pathogenesis and develop new strategies to combat infections, several studies investigated its virulence factors [11,18,40,41,42]. Remarkably, *A. baumannii* has a huge pathogenic potential that enables it to firmly resist in the environment, form biofilms, move, interact with host cells, capture micronutrients, and secrete proteins [3,13,16,18,41,42,43,44]. An overview of the virulence factors described in this review is presented in Table 1.

### 3.1. Survival Strategies 

The environmental survival of *A. baumannii* depends on its ability to persist against desiccation, disinfection and oxidative stresses. Besides other important functions, the biosynthesis of capsular polysaccharide and biofilm formation support the viability of *A. baumannii* in dry conditions for over three months [18,43,97,98]. The K locus encompasses clustered genes needed for the biosynthesis of the capsule [44,99]. Despite strain-specific biosynthetic genes accounting for the wide diversity of capsule composition, the K locus commonly includes genes encoding initiating transferases (*itr*), glycosyltransferases (*gtr*), assembly/export proteins (*wzx*/*wzy*), and modification enzymes (*atr*/*ptr*) [13]. The polysaccharides composing the capsule act as water retainers that shield the bacterial body against desiccation [100]; however, although mutant strains within the K locus displayed lower resistance, the heterogeneous behavior of encapsulated bacteria in relation to desiccation led to the conclusion that the capsule plays a minor role [100]. The lipid composition of the outer membrane, more specifically the acylated level of the lipo-oligosaccharide (LOS), has been suggested to be involved in resistance to dryness, in that the appropriate fluidity retains water and nutrients inside the bacterial cell [45,101]. Indeed, in *A. baumannii,* the common bacterial lipopolysaccharide is replaced with the LOS, which is composed of lipid A with variable amounts of inner and outer core sugars but lacks the O-antigen [45]. The LOS outer sugars show diversity across strains, dependent on glycosyltransferases and nucleotide-sugar biosynthesis enzymes encoded in a highly variable outer core locus [13]. 

The ability to form biofilms extends the desiccation tolerance of *A. baumannii* under dryness [100]. Biofilm formation starts with bacterial adherence to a surface and it is further strengthened by bacterial attachment and aggregation. These sessile bacterial communities are surrounded by a blended matrix consisting of DNA, exopolysaccharides (capsular and non-capsular) and proteins which protect pathogens from desiccation, since the exopolysaccharide matrix can retain water [100]. The Csu pili or Csu fimbriae are encoded by six-segmented operon, *csuA/BABCDE*, and are assembled by the chaperone–usher (CU) pathway [46]. Together with the biofilm-associated proteins (Bap), Csu pili are crucial for the formation and maintenance of biofilms on abiotic surfaces [11,16,18,46,47,102]. Some recent reviews describe the detailed network controlling biofilm formation [11,18,44,103,104,105]. Other important factors involved in biofilm formation are an effector protein encompassing a Repeats-in-Toxin (RTX)-like domain, poly-β (1-6)-N-acetyl-glucosamine (PNAG), the capsule, and autotransporter systems that will be discussed below [3,11,12,13,48]. Despite the remarkable desiccation tolerance of *A. baumannii* during environmental persistence, the restricted water availability could lead to DNA damages, osmotic and oxidative stresses [18,44,100]. *A. baumannii* copes with DNA damages, comprising alkylation, base omission, cross-linking, oxidation and strand breaks, with the RecA protein, which is recognized as an essential enzyme for homologous recombination [18,49,106]. Oxidative stress can be overcome by overexpression of genes encoding catalases. Indeed, *A. baumannii* is a catalase-positive bacterium; despite the genomic presence of four catalase genes, *katA*, *katE*, *katG*, and *katX,* only KatG and KatE defend the bacterium from oxidative stress [50,100]. Interestingly, both *katG* and *katE* genes are overexpressed through the upstream insertion of IS*Aba1* promoter sequences, thereby providing full protection from hydrogen peroxide (H_2_O_2_) [50,100]. Moreover, DNA damages as consequence of water stress, as well as other environmental stresses in clinical settings, generate base-pair substitutions in a number of different bacterial targets, thereby contributing to the acquisition of additional antibiotic resistances in *A. baumannii* (i.e., rifampin-resistance) [7,18,107]. Conversely, the osmoregulatory mechanism adopted by *A. baumannii* aims to increase the uptake of glycine betaine or synthesize glutamate and mannitol; without interfering with the general bacterial metabolism, these compounds, known as compatible solutes, stabilize proteins and membranes, thereby protecting bacteria from cell damage [18,100]. Osmotic stress, desiccation and resistance to antibiotics and disinfectants also involve efflux pumps, three-component protein systems (an outer membrane channel, a periplasmic lipoprotein, and an inner membrane transporter) that extrude disturbing or toxic molecules/compounds from inside the cell to the extracellular milieu. *A. baumannii* possesses several classes of efflux pumps, including major facilitator superfamily (MFS), resistance nodulation-division (RND), small multidrug resistance (SMR) family, and multidrug and toxic efflux (MATE), ATP binding cassette (ABC), and the proteobacterial antimicrobial compound efflux (PACE) family [53,55,108,109]. Apart from their role in antibiotic resistance, the RDN AdeABC, the MFS EmrAB efflux pumps as well as the RND-type AbeD transporter contribute to osmotic stress resistance [52,53,54,100,109]. Moreover, AbuO, a TolC-like protein, is involved in the oxidative stress response [54,109]. AmvA and AceI, belonging to the MFS and PACE efflux pumps respectively, were shown to be associated with the extrusion of disinfectants [54,55,56,109,110]. It is worth mentioning that the great adaptability of *A. baumannii* to such a variety of different stressful environments relies on several additional proteins that guarantee cellular homeostasis, such as GroEL, GroES, DnaJ, DnaK, ClpX, ClpB, OxyR, as well as Lon protease and other numerous chaperones [111,112,113,114]. Complex regulatory networks overlook these stress defense mechanisms, including two-component systems, the second messenger cyclic-di-GMP, the RNA chaperone Hfq, alternative sigma factors of the general stress response, and quorum sensing (QS) regulators [51,54,100,103,105,109,110,115]. 

### 3.2. Sensing the Quorum

Bacterial QS is a cell-to-cell communication system based on specific signaling molecules called ‘auto-inducers’ that allow bacteria to sense population densities. QS systems have a crucial role in the expression of virulence factors, motility, conjugation, biofilm formation and interactions with eukaryotic host cells [16,104,116,117,118]. To date, only one QS system was found in *A. baumannii*, consisting of the two *abaI* and *abaR* genes, acquired from *Halothiobacillus neapolitanus* through horizontal gene transfer. AbaI is the autoinducer synthase and AbaR is its cognate receptor, which belong to the typical LuxI/LuxR family members found in other Gram-negative bacteria. AbaI synthesizes N-(3-hydroxydodecanoyl)-1-homoserine lactone (Acyl Homo-serine Lactones, AHLs); upon binding of AHL to AbaR, the complex recognizes *lux*-box sequences on QS target promoters, thereby regulating their expression [57,119]. Indeed, QS molecules affect *A. baumannii bfmS* and *bfmR* genes by upregulating their expression leading to strong biofilm formation on abiotic surfaces [16,58]. Limited concentrations of iron also upregulate QS signaling molecules, enhancing *A. baumannii* persistence and virulence [16,119,120]. 

### 3.3. The Power of Pilus Retraction

Despite the name of this genus, *A. baumannii* is able to perform twitching motility through type IV pili. Encoded by the *pil* operon genes, these pili are cytoplasmic ATPase dependent projections that extend and retract to keep the bacterium going, mainly on wet surfaces [102]. It has been shown that the C-terminus of the major subunit of type IV pili, PilA, is glycosylated by O-oligo-saccharyl-transferases [59]. Interestingly, there is a high degree of variability both in amino acid sequence as well as in glycosylation of PilA proteins among *A. baumannii* isolates, most probably to evade the host immune system [121]. Although a clear link between twitching motility and virulence has not yet been established, several studies showed an upregulated biogenesis of type IV pili when grown in serum with respect to sputum, highly suggestive of a key role of twitching motility during bacteremia [13,18,44]. However, the role of type IV pili is not restricted to twitching motility but also to biofilm formation, virulence and DNA uptake [13,18,44,59]. Indeed, it has been reported that type IV pili promote host-cell adhesion to both pharynx and lung carcinoma cells in vitro [121]. In addition to twitching motility, some isolates of *A. baumannii* can move by surface-associated motility [13,18,44]. This motility seems to be independent from type IV pili and relies on the biosynthesis of polyamine 1,3-diaminopropane, LOS and QS [13,18,44]. Although the precise mechanism of surface-associated motility remains to be elucidated, the signaling network of cyclic-di-GMP, the second messenger molecule involved in adaptation to various stress responses, was shown to upregulate biofilm formation and downregulate surface associated motility [105]. 

### 3.4. Exploring Surface Proteins

Outer membrane proteins (Omps) embedded within the outer membrane (OM) are cornerstone proteins involved in cellular permeability and virulence in *A. baumannii*. These monomeric or trimeric β-barrel proteins, also known as porins, connect the external environment to the periplasmic space, allowing the diffusion of nutrients as well as small molecules, antibiotics, and disinfectants [18,40,41,44,51]. To date, identified Omps in *A. baumannii* are OmpA, CarO, OprD-like, Omp33-36 kDa, OmpW, AbuO, TolB, DcaP, Oma87/BamA, NmRmpM, CadF, OprF, LptD [18,41,44]. Besides its role in antibiotic-resistance, OmpA plays a central role in *A. baumannii* virulence, including, serum resistance, biofilm formation, host-interaction, cytotoxicity, and apoptosis [11,18,60,61,62,63,64,65,66,67,122]. OmpA has eight- antiparallel β-barrel strands embedded within the OM at the N-terminus while the C-terminus is bound to a peptidoglycan-derived pentapeptide, thereby performing also a structural role [123]. The amino acid sequence of this protein is highly conserved among *A. baumannii* clinical isolates; its involvement in antibiotic-resistance was revealed by the use of *ompA* mutants [41,62]. However, the increase in antibiotic-susceptibility of these mutants could be related to a broader membrane permeability as a consequence of membrane alterations [61]. Nevertheless, several studies demonstrated that OmpA acts as a specific and selective channel for small antibiotics [41,61,62,124]. However, multiple data indicate that OmpA is associated with efflux pump systems located in the inner membrane to counteract the influx of antibiotics [62]. Due to its key role, several proteins control *ompA* expression such as the global repressor H-NS and an anti-repressor (i.e., gene locus A1S_0316), the RNA chaperone Hfq as well as the two-component system BfmSR [41,125,126]. Its high amino acid conservation among isolates, crucial structural role and strong immunogenicity make OmpA the ideal target for the development of an *A. baumannii* vaccine [124]. Additionally, several studies have addressed the role of CarO or carbapenem susceptibility porin in *A. baumannii* [13,41,68,69,70]. Limansky et al. first demonstrated that resistance to carbapenems among clinical isolates, specifically to imipenem, was due to the loss of CarO [127]. CarO is the channel for the transport of small amino acids such as glycine and ornithine, but it was shown to mediate the influx of imipenem into the bacterial cells [13,41]. While this may seem very harmful, CarO physically interacts with the most widespread carbapenemase OXA-23 in *A. baumannii* so that imipenem is hydrolyzed immediately upon entry into the bacterial periplasm [128]. Additionally, five porins orthologous to OprD from *P. aeruginosa* were identified in carbapenem-resistant *A. baumannii* isolates; they belong to the Occ class (OccAB1–5) [129]. Structural studies of OccAB1–4 showed that they are 18-stranded β-barrel proteins characterized by different pore diameters; OccAB1 has the largest channel and was the most efficient in the uptake of carbapenems [70,73,74,75,76,129]. Formerly known as porinD or OprD, this protein was showed to be involved in the transport of different molecules including amino acids, sugars and antibiotics such as meropenem and Fosfomycin [129]. Moreover, OprD together with OmpW were linked to iron uptake in *A. baumannii* [77]. OmpW is an eight-stranded OM β barrel protein that shares several features with OmpA; its amino acid sequence is highly conserved among *A. baumannii* isolates, it is greatly immunogenic, highly concentrated in OM vesicles (OMVs) and it has cytotoxic activity against host cells [63,77,130]. OMVs are micro-spherical vesicles of 20-200 nm in diameter that are secreted by the secretory system independently from conventional systems [11,42,63,96,131,132]. These vesicles are composed of LOS, Omps, phospholipids, periplasmic proteins, as well as DNA and RNA molecules; OMVs are a means through which bacteria deliver a number of bacterial virulence factors to other bacteria or host cells, thereby inducing host cell damage and innate immune responses [7,11,13,19,42,63]. Another important Omp in *A. baumannii* is Omp33-36 kDa or Omp34 or Omp33 [41,71,72]. Its resolved crystal structure showed that Omp33 has 14 antiparallel β-strands connected by 7 loops extending outside and 6 turns protruding into the periplasmic side; recently, it was reported that two of these periplasmic turns block the aqueous channel [41]. Interestingly, it was found that Omp33 plays an important role in fitness and virulence in *A. baumannii*; this cytotoxic protein triggers apoptosis via caspase activation while modulating autophagy to enhance its persistence within host cells [71,72]. 

### 3.5. Micronutrients Hunger

Micronutrients are fundamental for bacterial survival and growth. *A. baumannii* possesses different metal uptake systems for scavenging zinc, iron and manganese as well as other valuable and essential nutrients. Iron bioavailability is scarce both in the environment and in hosts. To acquire free iron, *A. baumannii* produces and secretes siderophores, low molecular weight iron scavengers (400–1000 kDa) able to chelate it at high affinity. The catechol-hydroxy-mate siderophores including acinetobactin, fimsbactins A-F, baumannoferrin A and B are examples of *A. baumannii* iron chelators [78]. However, acinetobactin is the most conserved and recognized siderophore in *A. baumannii* [7,11,18,133]. According to genomic studies, bacterial siderophore biosynthetic genes are usually clustered and under the transcriptional control of the ferric uptake regulator Fur encoded by the *fur* gene [11,79,114]. Due to the importance of iron in *A. baumannii* physiology, the expression of a huge number of genes is under the control of iron availability, including those encoding for efflux pumps belonging to MFS, MATE, and ABC families, QS, Bap, phospholipases C and D, catalase and superoxide dismutase [51,54,55,80,81,108,109,110,134]. Indeed, to overcome the high reactivity of iron via the Fenton reaction, iron acquisition and metabolism is associated with the expression of OxyR and SoxR, responsible for reactive oxygen species (ROS) detoxification and for super oxide response, respectively [11,18,82,83,111,135,136]. *A. baumannii* phospholipases C and D are potent virulence factors characterized by hydrolytic and lipolytic activities; these enzymes showed hemolytic activities by targeting red blood cells in order to provide iron for *A. baumannii* growth during the infection process [7,11,18,133,136]. In addition, iron receptors or transport proteins that bind directly ferrous (Fe^2+^) ions are located on the bacterial cell surface [11]. In addition to iron, zinc (Zn) and manganese (Mn) are important micronutrients for growth and virulence in *A. baumannii*. Being a cofactor of metalloproteins, e.g., metalloproteases, *A. baumannii* employs an ABC transporter system, ZnuABC that works together with the OM TonB-dependent receptor ZnuD for the uptake of zinc; the whole system is under the control of the transcriptional zinc uptake regulator Zur [84,137]. Thereafter, the Zn metallochaperone ZigA seems to assist the transfer of the precious metal to metalloproteins, although more studies are required to elucidate the precise mechanism [11,18,85,136]. Mn is the redox-active cofactor for enzymes that protect the bacteria from ROS, such as superoxide dismutase and ribonucleotide reductase [11,42]. Like other pathogens, *A. baumannii* has a high-affinity Mn transmembrane transporter, MumT, belonging to the resistance-associated macrophage protein (NRAMP) family, that uses the proton motive force as an energy source for the uptake of exogenous manganese [86,111,138]. Being involved in several biochemical reactions, phosphate has two dedicated transport systems in *A. baumannii*, a low- and a high-affinity transport system, encoded by the *pit* gene and the *pst* operon (*pstA*, *pstB, pstC, pstS*, and *phoU*), respectively [87,88]. In this latter, the periplasmic phosphate-binding protein PstS controls the system by sensing phosphate levels and by transferring it to its specific transporter; the whole operon is transcriptionally activated by the two-component system PhoB/R under phosphate deficiency [89,139]. Interestingly, it was recently reported that PstS plays an important role in *A. baumannii* virulence during microaerobic conditions; indeed, the *pstS* deletion mutant showed reduced adhesion to and invasion of human alveolar type II cells (A549 cell line, see below), whereas its overexpression enhances pathogenesis [88]. Supported also by in vivo results, it was concluded that PstS is important for *A. baumannii* pathogenicity and spread within the host [88].

### 3.6. The Versatility of Secretion Systems

Bacteria secrete proteins to adapt more easily to environmental conditions and host-interactions. To date, five secretion systems have been identified in *A. baumannii*, comprising type 1 secretion system (T1SS), T2SS, T4SS, T5SS, and T6SS [11,18,44]. T1SS is composed by an inner membrane (IM) ATPase protein (hemolysin secretion protein B, HlyB), a periplasmic adaptor (hemolysin secretion protein D, HlyD), and an Omp (TolC); this system contributes to exporting proteins involved in biofilm formation and maintenance and assisting also to adhesion with pulmonary epithelia, i.e., Bap and Blp1, as well as the RTX-serra-lysin-like toxin [12,140]. Conversely, the T2SS involves 12–15 proteins including cytoplasmic ATPases, IM platform assembly, OM secretins and periplasmic pseudo-pili; the pseudo-pilus works as a sort of piston that extrudes substrates out of the bacterial cells though OM secretins. Genes encoding the proteins composing T2SS are referred to as general secretory pathway (Gsp) [90,141]. T2SS accommodates proteins with an export signal from the Sec or the Twin-arginine (Tat) translocons before delivering them outside the cell; important virulence factors such as lipases (LipA and LipH), zinc-dependent metallo-endopeptidase (CpaA), elastase, alkaline phosphatase, and phospholipases are secreted by T2SS [3,13]. Interestingly, T2SS and type IV pili share the pilin peptidase PilD/GspO that processes pre-pseudo-pilins and pre-pilins before assembly into the T2SS and type IV pilus, respectively, revealing their evolutionary relatedness [141,142]. T4SS (type F) is devoted to conjugative transfer of DNA, plasmids, and other mobile genetic elements; therefore, this system is thought to be responsible for the spread of antibiotic-resistance genes among *A. baumannii* clinical isolates and particularly OXA- 23 [44,91,143]. Conversely, the T5SS, also known as autotransporters, represents the simplest and most widespread secretion system in Gram-negative bacteria; the name is due to the ability of these proteins to cross the OM autonomously [144,145]. T5SS is composed of five subgroups, T5aSS, T5bSS, T5cSS, T5dSS and T5eSS [145]. This classification is based on the specific features of the passenger/translocation domains, quaternary structure (i.e., monomeric or trimeric), and the terminal residues of proteins exposed on the surface (N- or C-terminus) [145]. Only the T5bSS (AbFhaB/C and CdiA/B) and T5cSS (Ata) subgroups were found in *A. baumannii* [11,92,93]. In the T5bSS subclass, also termed the two-partner secretion (TPS) system, passenger and translocation domains (TpsA and TpsB, respectively) are allocated onto two distinct proteins whose genes are transcriptionally linked [11]. The 16-stranded β barrel AbFhaC protein recognizes and translocates to the cell surface its partner protein, AbFhaB, via two specific periplasmic polypeptide transport-associated domains; AbFhaB has the arginine-glycine-aspartic acid (RGD) motif that was shown to be associated with eukaryotic integrin and fibronectin attachment [92]. The contact-dependent inhibition (CDI) systems are strategies that *A. baumannii* uses to kill bacterial competitors; the OM pore CdiB allows the secretion of the CdiA toxin that kills bacterial cells that do not have the antitoxin or the immunity protein CdiI [3]. *A. baumannii* has two CDI loci, Cdi1 and Cdi2, each composed of the transporter, the toxin and the immunity proteins [42]. On the other hand, *Acinetobacter* trimeric autotransporter, or Ata, belongs to the T5cSS; each monomer of this large homo-trimeric protein consists of a C-terminal domain that forms a 4 β-strand hydrophilic pore within the OM through which it extrudes the N-terminal passenger domain of each monomer [94]. The passenger domain contains four pentameric collagen binding consensus sequences (SVAIG) and one RGD motif able to bind extracellular matrix and basal proteins (i.e., collagen I, III, IV, and V and laminin) and to participate to biofilm formation [94]. Finally, several *A. baumannii* strains carry a genetic locus for T6SS; this contact-dependent multi-component apparatus is encoded by 13 core structural proteins together with accessory (i.e., TagF, TagN, PAAR, and TagX) and regulatory proteins (i.e., VgrG1, TetR) [95]. Indeed, due to the high-demanding energy costs, T6SS is activated upon stressful stimuli, including nutrient limitation, cell damage and competing bacteria [146]. Accordingly, the T6SS enables *A. baumannii* to inject into other bacteria toxic proteins, including peptidoglycan hydrolases, nucleases, or those effectors targeting the cell membrane, to outcompete neighboring bacteria [147]. 

It is important to note that in the past, *A. baumannii* was considered a commensal, and a relatively low-grade opportunistic pathogen; the main features of *A. baumannii* virulence factors highlighted the great pathogenic potential of this bacterium. Nevertheless, it should be taken into account that the immune status of the human host plays a big role in the infective success of *A. baumannii.* In the following paragraphs, the components of *A. baumannii* virulome important for the interaction with host cells and evasion of the host immune response will be discussed. 

## 4. Host-*A. baumannii* Interactions: The Respiratory Epithelium

**Figure 1:**
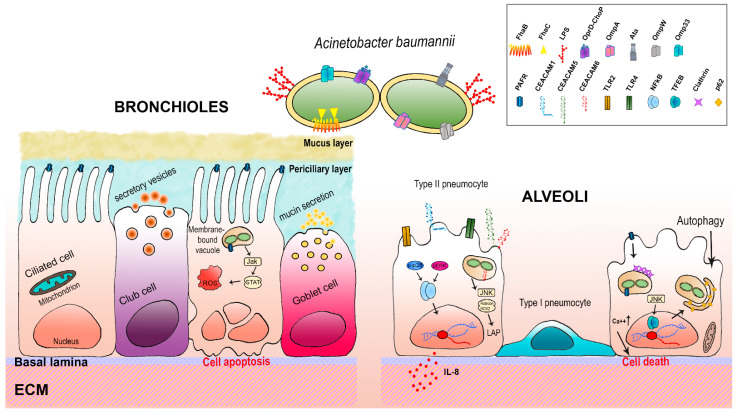
Schematic representation of the interrelations between *A. baumannii* and the respiratory epithelium. *A. baumannii* has several virulence factors that enable the bacterium to adhere to and invade host cells. Main proteins involved in the interaction with host cells and extracellular host proteins are OmpA, Omp33, OmpW, Ata and FhaB. *A. baumannii* cells are internalized via a zipper mechanism and reside and survive within membrane-bound vacuoles. Bronchial epithelial cells respond to *A. baumannii* infections by eliciting the Jak-STAT pathway as well as the intracellular oxidative stress response that eventually lead to apoptosis [169]. TLR2 and TLR4 on type II pneumocytes recognize lipoproteins and LOS and release cytokines (i.e., IL-8) via NF-κB and p38-Erk1/2-dependent pathway to chemoattract neutrophils at the site of infection [163]. *A. baumannii* can engage CEACAM-1, -5, and -6 to gain access to type II pneumocytes. Internalization through CEACAM-1 triggers IL-8 production together with TLR2 and TLR4 via NF-κB and Erk1/2-dependent pathway; however, IL-8 secretion decreases significantly at 24 h post-infection, possibly due to a bacterial-induced inhibitory activity of CEACAM-1 ITIM on the TLR2 signaling cascade. Conversely, engagement of CEACAM-5 and -6 triggers induce LC3 associated phagocytosis (LAP) for clearance of *A. baumannii* via the JNK1/2-Rubicon-NOX2 pathway, which inhibits the canonical autophagic pathway [170]. Furthermore, *A. baumannii* can interact with platelet-activating factor receptors (PAFRs) via ChoP-containing OprD, leading to a signaling cascade that includes G protein-coupled PLC, clathrin, β-arrestins and an increase of intracellular Ca^++^, thereby leading to bacterial internalization [76]. Invasion and persistence of *A. baumannii* is assisted by TFEB which induces the autophagosome-lysosome system that *A. baumannii* exploits to traffic intracellularly and persist within lung cells, possibly due to reduced lysosome acidification [150]. It has been hypothesized that both intracellular persistence and apoptosis are *A. baumannii* strategies to allow bacterial dissemination to deeper tissues and lead to invasive diseases. Individual components are not to scale.

Studies on the virulence of *A. baumannii* have grown as a consequence of the worrisome rise in the number of antibiotic-resistant isolates. Due to the high prevalence of ventilator-associated pneumonia, most researches focused on in vitro and in vivo pulmonary models [11,13,18,19]. The respiratory mucosa includes the epithelium, the basal lamina and lamina propria; these latter two represent the extracellular matrix (ECM) and provide structural and biochemical support to surrounding cells. The ECM is composed of a huge number of different proteins, including collagen, elastin, fibrillin, laminin, fibronectin, and vitronectin [148]. Instead, the alveolar epithelium is structured by alveolar type I and type II cells; type I cells are responsible for gas exchange together with the underlying endothelium, whereas production of surfactant proteins and protection against airborne pathogens is carried out by type II cells [149]. The receptors expressed by alveolar cells as well as the ECM proteins represent adhesive surfaces for bacterial adhesins; these surfaces allow the establishment of physical contact between host cells and pathogenic bacteria such as *A. baumannii* [11]. The first evidence that *A. baumannii* can adhere to and invade host epithelial cells was provided by Choi et al. [65]. Using in vitro assays, it was shown that *A. baumannii* is internalized by a zipper-like mechanism that involves a cellular receptor; once internalized, bacterial cells reside within membrane-bound vacuoles [65]. Choi et al. highlighted the primary contribution of the major porin OmpA, which was the focus of several following studies [11,13,41,60,65,66,150]. Indeed, it was shown that OmpA interferes with cell autophagy [66,150]. Autophagy is a degradative process that clears damaged cytoplasmic proteins and organelles as well as some phagocyted bacteria by delivering them to lysosomes [151]. This complex process requires several proteins including autophagy-related proteins (ATGs), beclin 1, microtubule-associated protein light chain 3 (LC3), Ras related protein (Rab)-5, Rab-7, and p62 [151]. An et al. have shown that *A. baumannii* OmpA triggers autophagy in both epithelial and macrophage cell lines (i.e., Hela and RAW264.7, respectively) via activation of the mitogen-activated protein kinase (MAPK) c-Jun N-terminal kinase (JNK) signaling pathway; they reported a rise in the lipidated form of LC3 (LC3BII) and a block in p62 degradation [66]. In addition, OmpA prevents the fusion between autophagosomes and lysosomes, thereby causing an incomplete autophagy that allows survival and persistence of *A. baumannii* bacterial cells within the autophagosomes [66]. As a consequence of incomplete autophagy, cells enhance the release of inflammatory cytokine IL-1β, inducing a systemic inflammatory response that possibly allows bacteria to spread to adjacent tissues [66]. These observations were further corroborated and extended also to porin Omp33 [60,71,72,76,150]. An important feature of *A. baumannii* during the infective process is the release of OMVs, composed of OmpA and Omp33; it has been reported that internalized OmpA concentrates via activation of GTPase dynamin-related protein 1 within host mitochondria where it causes mitochondrial fragmentation, an increased production of ROS and, eventually, cell death, both in vitro and in vivo models [63]. Adhesion to host cells was also shown by the T5cSS Ata protein; Ata binds to collagen I, III, IV and V and laminin molecules via an RGD motif and four SVAIG [11]. Indeed, *ata* mutants displayed an attenuated virulent phenotype in in vivo models [11]. Another host structure exploited by adhering bacteria is the ECM; it interacts with epithelial cells through cellular adhesion molecules, such as integrins, immunoglobulin cell adhesion molecule superfamily (IgCAMs), selectins, and cadherins [152]. Interestingly, the *A. baumannii* T5bSS FhaB protein binds both host integrins and fibronectins, having a RGD motif [11,92,153]. The presence of this domain in *A. baumannii* adhesin is due to the fact that the RGD motif is widespread among ECM proteins, including laminin, collagen I and fibronectin, being specifically recognized and bound by the majority of integrins [154]. Consequently, the binding between integrins and particular bacterial adhesins induces intracellular signaling pathways within host cells that lead to reorganization of the actin cytoskeleton which results in bacterial internalization [155,156]. A schematic representation of *A. baumannii*-host interactions herein described can be seen in Figure 1.

Host cell receptors represent an anchoring handle for bacterial adhesins, through which bacteria establish physical contact, mediate internalization via zipper mechanisms and trigger host responses [157,158,159]. To study the role of receptors in host-pathogen interactions in pulmonary in vitro models, the A549 cell line, human type II epithelial cells from lung adenocarcinoma, has been extensively used [60,76,150,160,161]. Like other epithelial cells, pulmonary cells expose a number of surface receptors, including Toll-like receptors (TLRs), specific carcinoembryonic antigen-related cell adhesion molecules (CEACAMs), as well as platelet-activating factor receptors (PAFRs). 

### 4.1. TLRs

TLRs are sentinels for the detection of and response to microbial infection by recognizing specific bacterial components or pathogen-associated molecular patterns (PAMPs) [162]. Thus, upon recognition of their specific microbial components, TLRs activate signaling pathways aimed at killing pathogens [162]. Among the 10 human TLRs identified so far, TLR2 binds to bacterial lipoproteins, TLR4 recognizes lipopolysaccharide (LPS), and TLR9 is activated by unmethylated CpG containing ssDNA [162]. Using the A549 cell line experimental model, March et al. showed that *A. baumannii* is recognized by both TLR2 and TLR4 which, upon binding, trigger interleukin-8 (IL-8) secretion via MAPKs p38 and extracellular signal-regulated kinase (ERK)1/2 through nuclear factor-kappa B (NF-κB) activation [163]. Secretion of IL-8 is critical for lung influx of neutrophils for *A. baumannii* clearance, both in vitro and in vivo [163,164,165]. Differently from other TLRs, TLR9 is located intracellularly in endosomes. Its involvement in *A. baumannii* infections was found by the use of *tlr9* -/- knockout mice; infected animals developed more severe lung lesions as well as greater extra-pulmonary bacterial dissemination compared to wild-type mice [166]. Interestingly, the same effects were observed using an animal model of aged mice, proficient in TLR9; it was concluded that the expression of TLR9 is particularly important for the immune response of elderly people against *A. baumannii* lung infection [167]. 

### 4.2. PAFRs

Another class of surface-exposed receptor is the PAFR distributed on the epithelial cells of many tissues, including the respiratory system; this G-protein-coupled seven-transmembrane receptor recognizes physiologically the pro-inflammatory mediator platelet-activating factor (PAF), a potent phospholipid mediator [168]. However, since phosphorylcholine (ChoP) moieties are shared with PAF, PAFR binds to bacterial ChoP, thereby allowing bacterial adhesion to and invasion of the respiratory epithelium [156]. As with other respiratory pathogens, *A. baumannii* carries ChoP-containing OprD to exploit the ChoP-PAFR strategy to adhere to lung epithelial cells [76]. In addition, Smani et al. demonstrated that binding to PAFR triggers an endocytic pathway, involving G-protein-phospholipase C, β-arrestins and clathrin, that leads to the internalization of *A. baumannii* into lung epithelial cells both in vitro and in vivo [76]. Moreover, using the A549 cell line, Parra-Millán et al. demonstrated that *A. baumannii* infections activate the transcription factor EB (TFEB) [150]. TFEB leads to upregulation of several autophagic genes (up to 79), including LC3BII which in cooperation with p62 induces the autophagosome-lysosome system that *A. baumannii* exploits to traffic intracellularly and persist within lung cells, possibly due to reduced lysosome acidification [150]. More recently, it was reported that *A. baumannii* ChoP-PAFR-mediated entry of human bronchial epithelial cells can also elicit the Janus kinase (Jak)-signal transducer and the activator of transcription (STAT) pathway, as well as intracellular oxidative stress and apoptosis as a response to the bacterial infections [169]. 

### 4.3. CEACAMs

Some respiratory pathogens adhere to CEACAM receptors, a group of immunoglobulin (Ig)-related glycoproteins that are involved in several cellular processes such as cell adhesion, intracellular and intercellular signaling, inflammation, and cancer progression [156,171,172]. These highly glycosylated proteins consist of an immunoglobulin (Ig) variable domain (IgV) on the top of its exterior portion followed by zero to six Ig constant domains (IgC). The IgV domain is recognized by the several pathogenic Gram-negative bacteria [156]. Among the members of mammalian CEACAMs, epithelial cells express CEACAM-1, -5 and -6 on their surface [171]. Differently from CEACAM-5 and -6 which are associated with the membrane through a glycosylphosphatidylinositol (GPI) lipid moiety, CEACAM-1 is composed of four extracellular Ig-like domains, a transmembrane region and the cytoplasmic domain containing an immunoreceptor tyrosine-based inhibitory motif (ITIM) [171]. Recently, it was shown that *A. baumannii* adheres to and is internalized into host lung epithelial cells through the interaction with CEACAM-1, CEACAM-5 and CEACAM-6 [170]. Engagement of these receptors by *A. baumannii* triggers two different CEACAM-dependent signaling pathways. The CEACAM-1-dependent pathway initially leads to IL-8 secretion via Erk1/2 and NF-κB signaling; IL-8 levels drop dramatically after 24 h of infection, possibly due to an *A. baumannii*-dependent effect on the CEACAM-1 intracellular domain. Conversely, CEACAM-5 and -6 trigger LC3 associated phagocytosis (LAP). Upon phagocytosis, this pathway recruits autophagic proteins for a non-canonical function to promote the fusion of phagosome with lysosomes, to enhance bacterial killing. Accordingly, it was demonstrated that LAP eliminates *A. baumannii* bacteria cells through the pathway of JNK1/2-Rubicon-nicotinamide adenine dinucleotide phosphate oxidase 2 (NOX2) that eventually leads to phago-lysosome fusion [170]. Overall, we have started learning about the complex processes occurring at the interface between *A. baumannii* and the pulmonary cells; however, considerable gaps need to be filled in. The rapid progress in in vitro cell cultures, model organisms and animal models will expand our knowledge about the molecular interactions of *A. baumannii* in the course of lung infections.

## 5. How *A. baumannii* Fights Host Immune Attacks 

**Figure 2:**
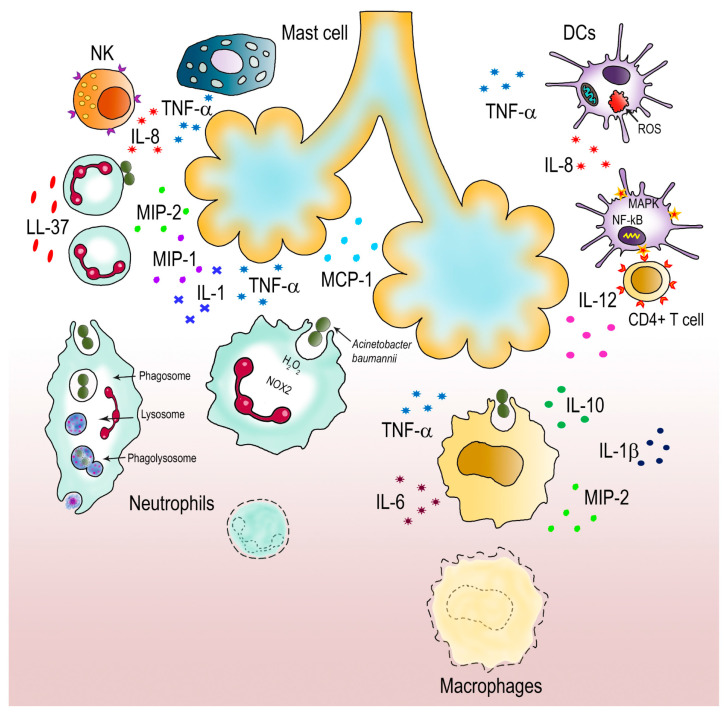
Innate immune responses to *A. baumannii* in respiratory epithelia. Neutrophils, macrophages, mast cells, NK and DC cells are involved in the clearance of *A. baumannii* infections. Neutrophils are the main defense against *A. baumannii* infections [165]. Neutrophils are depicted during phagocytosis and pathogen clearance. Massive recruitment of neutrophils is achieved by secretion of antimicrobial peptides as well as chemoattractant cytokines and chemokines, including interleukin-1 (IL-1), macrophage inflammatory protein-1 (MIP-1), MIP-2, monocyte chemoattractant protein-1 (MCP-1), tumor necrosis factor (TNF-α) [11,19,175]. However, *A. baumannii* has developed several strategies to evade neutrophil killing activities. Invading *A. baumannii* can inhibit the production of NETs; in addition, bacteria can adhere to IL-8-activated neutrophils and use them as transporters in vitro [165,176]. Moreover, phagocyted *A. baumannii* can oppose the killing effects of ROS produced by neutrophils by upregulating bacterial resistance compounds [138]. While phagocyting a significant number of *A. baumannii* cells, macrophages release proinflammatory cytokines and chemokines, such as IL-6, IL-10, IL-1β, MIP-2 and TNF-α, in order to recruit neutrophils at the site of infection [11,175]. DCs join the innate immune system into the adaptive immune system by producing IL-12 to induce CD4+ Th1 T cell immune responses [175]. Despite that *A. baumannii* OmpA can activate dendritic cells (DCs), it induces death of DCs via mitochondrial targeting and ROS production, thereby impairing the adaptive immune responses [175]. It seems that the main role of the N-terminal kinase (NK) and mast cells against *A. baumannii* is to produce neutrophil chemo-attractants, such as IL-8 and TNF-α [11,175]. For simplicity, the toll-like receptors (TLRs) signaling pathways were omitted. Individual components are not to scale.

The innate immune system is known to be the first defense line against bacterial invaders. Phagocytes, such as neutrophils, macrophages, mast cells, natural killer (NK) and dendritic cells (DCs) are the main responders of this system [11,173,174]. Together with epithelial cells, these innate immune cells express two groups of pattern recognition receptors (PRRs), TLRs and nucleotide-binding oligomerization domain (NOD) receptors, both involved in *A. baumannii* recognition [42,175]. These receptors recognize specifically PAMP components (i.e., LOS, OMVs, capsule) and damage-associated molecular patterns (DAMPs) that are released upon infection from damaged tissues or injured host cells [42,175]. As previously described, TLRs have a relevant role in the induction of different TLR signaling pathways against *A. baumannii*. [11,175]. A schematic representation of the innate immune response to *A. baumannii* infections is provided in Figure 2.

A broad body of evidence has underlined the role of neutrophils in the pulmonary response to *A. baumannii* infection. Their importance was demonstrated by the use of neutropenic animals; in this model, the lack of neutrophil recruitment as well as related chemokines and cytokines allowed higher lung bacterial burden and led to acute lethal infections [177]. Indeed, neutrophils are quickly chemoattracted to *A. baumannii* infection sites, reaching the highest number after 24 h [11]. During infections, neutrophils use several bactericidal weapons, including degranulation, phagocytosis, oxidative burst and neutrophil extracellular traps (NETs) [165]. NETs are molecular fibers composed by DNA plus a vast number of proteins aimed at killing bacteria [178]. However, *A. baumannii* has the ability to inhibit NET formation even in the presence of neutrophil activators (e.g., IL-8, phorbol-myristate acetate or LPS); additionally, *A. baumannii* cells can adhere to IL-8-activated neutrophils and use them as transporters in vitro [165,176]. On the other hand, it was shown that oxidative burst is an effective bactericidal strategy used by neutrophils to kill *A. baumannii*; this mechanism relies on the production of ROS, mainly by NOX2 [19,175]. As mentioned earlier, by detoxifying H_2_O_2_ into water and oxygen, KatG and KatE are able to attenuate the neutrophil-mediated oxidative injury [179]. It is important to underline that *A. baumannii* MumR, involved in the Mn transport system, confers resistance to ROS from neutrophils, due to its involvement in the regulation of phenylacetate and gamma-aminobutyric acid catabolism pathways [138]. These compounds considerably increase *A. baumannii* resistance under oxidic conditions, thereby representing an additional defense mechanism that this bacterium uses against neutrophil attacks [138]. Moreover, antimicrobial peptides secreted by both neutrophils as well as epithelial cells showed important roles during *A. baumannii* infections; in vivo models showed that the soluble factor cathelicidin LL-37 released by neutrophils has both antibacterial and neutrophil chemoattractant activities [19]. Therefore, massive neutrophil recruitment as well as the production of chemoattractant cytokines and chemokines, including interleukin-1 (IL-1), macrophage inflammatory protein-1 (MIP-1), MIP-2, monocyte chemoattractant protein-1 (MCP-1), and tumor necrosis factor-α (TNF-α) seem to be essential for the effective killing of *A. baumannii* [19,175]. It is worth mentioning that the bactericidal mechanism of phagocytosis by neutrophils is in association with the TLR signaling pathway activity, complement system and IgG opsonization and it occurs in a short and limited time duration of ~20 s, involving ROS, defensins, lysosomes and granular fusion mechanisms [11,18,19,173,175].

Macrophages are other immune cells that have key role in controlling infections caused by *A. baumannii*. Together with monocytes, macrophages release chemokines for local recruitment of neutrophils during the early phases of *A. baumannii* infections. Data based on in vivo models showed that the initial activity of macrophages is crucial for the control of the progression of the disease [19]. Indeed, macrophages are able to phagocyte a significant number (>80%) of *A. baumannii* bacterial cells [19,175]. At the same time, they release proinflammatory cytokines and chemokines, such as IL-6, IL-10, IL-1β, MIP-2 and TNF-α, in order to recruit neutrophils at the site of infection [11,175]. Despite being less effective than neutrophils, the importance of macrophages during *A. baumannii* infection was demonstrate in in vivo experiments; indeed, animals depleted of macrophages showed a dramatic increase of the bacterial burden at 24 h post-infection, thereby increasing animal susceptibility to *A. baumannii* infection [11,19,175]. Recently, a sex-based innate immune response to *A*. *baumannii* respiratory tract infection was reported; the higher susceptibility of female mice to *A. baumannii* infection was correlated to a reduction of both neutrophils and alveolar macrophages [180]. In female mice, the enhanced inflammation is linked to a delay in the recruitment of the immune cell population, thereby affecting their ability to immunologically respond efficiently to *A. baumannii* infections [180]. 

Other innate immune cells, such as DCs, NKs, and mast cells also contribute to the innate immunity against *A. baumannii* infections. Accordingly, it was reported that *A. baumannii* OmpA is able also to activate DCs. As antigen presenting cells, DCs are capable of joining the innate immune system into the adaptive immune system. Therefore, via activation of MAPKs and NFκB, DCs produce IL-12 to induce CD4+ Th1 T cell immune responses [175]. However, despite activating DCs, OmpA can induce DC death via mitochondrial targeting and ROS production, thereby impairing adaptive immune responses against *A. baumannii* infections [175]. NK cells were shown to be involved in the defense against *A. baumannii*; indeed, NK-depleted mice showed decreased survival rates and a stable bacterial burden [11,175]. However, NK cells contribute indirectly to *A. baumannii* clearance by producing neutrophil chemo-attractants, comprising IL-8 and TNF-α [11,175]. Therefore, it is believed that the decreased survival of *A. baumannii* infected animals is mostly due to an inefficient recruitment of innate immune cells to infection sites [11,175]. Mast cells also are involved in host defense against pathogens. Being located on epithelial and mucosal surfaces, these cells initiate an immune response against *A. baumannii* by producing IL-8 and TNF-α to recruit and activate neutrophils at the site of infection. Although it is highly evident that the innate immune cells play a critical role in fighting *A. baumannii*, at present we need to fill several gaps in the specific and interconnected roles of each of them during *A. baumannii* infections. In addition, the adaptive immunity response against *A. baumannii* infection is an even less explored area. Despite the fact that several proposed vaccine candidates targeting *A. baumannii* have been identified, further research will definitely be needed to exploit these for immune-based therapies and effective vaccination strategies. 

## 6. Conclusions

Genomic studies of *A. baumannii* have revealed the high degree of heterogeneity among strains belonging to this species due to its fearsome genomic plasticity. However, these studies suggested a distinction between environmental and nosocomial strains; strains belonging to this latter group showed a further differentiation depending on whether they interacted with hosts or with the hospital environment. It seems that this evolution process impacts dramatically on public health. In the past, *A. baumannii* was considered a pathogen endowed with low virulence; today we should look at it as a pathogen carrying an arsenal of virulence factors, which can be further acquired and shared by a constant adaptive evolution process triggered by environmental/host stimuli. Due to the rise in the number of pan-drug-resistant strains against which all our antibiotic classes are blunted weapons, it is of utmost importance that research focuses on the complex interplay between the host and microorganisms as well as the mechanisms *A. baumannii* use to evade host immune defenses. Understanding the molecular mechanisms that drive *A. baumannii* pathogenicity is crucial for the development of anti-adhesion and anti-virulence strategies. Interfering with bacterial adhesiveness to host proteins, virulence and cell-to-cell signaling pathways is a promising approach to defeating *A. baumannii* strains, towards which available antibiotic therapies are ineffective and even the pharmaceutical industry has surrendered.

## Figures and Tables

**Table 1:** Overview of *A. baumannii* virulence factors reported in this review.

Gene(s)	Virulence Factor(s)	Function(s)	Reference
K locus	capsule	Persistence	[44]
OC locus	Lipo-oligosaccharide (LOS)	Dryness resistance	[45]
*csuA/BABCDE*	Csu pili	Biofilm formation	[46]
*bap*	Bap	Biofilm formation	[47]
M215_09430 locus	Repeats-in-Toxin (RTX)-like domain	Biofilm formation	[12]
*pgaABCD* locus	PNAG	Biofilm formation	[48]
*recA*	RecA	DNA damage repair	[49]
*katG*	KatG	Oxidative stress resistance	[50]
*katE*	KatE	Oxidative stress resistance	[50]
*abuO*	AbuO (component of an ABC efflux pump)	Oxidative stress response	[51]
*adeABC*	AdeABC (RDN efflux pump)	Osmotic stress resistance	[52]
*emrAB*	EmrAB (MFS efflux pump)	Osmotic stress resistance	[53]
*abeD*	AbeD (component of an RND-type efflux pump)	Osmotic stress resistance	[54]
*amvA*	AmvA (component of an MFS efflux pump)	Resistance to disinfectants	[55]
*aceI*	AceI (component of a PACE efflux pump)	Resistance to disinfectants	[56]
*abaI*	AbaI (component of the QS system)	Virulence, motility, conjugation, biofilm formation and host-pathogen interactions	[57]
*abaR*	AbaR (component of the QS system)	Virulence, motility, conjugation, biofilm formation and host-pathogen interactions	[57]
*bfmS*	BfmS	QS-regulated two-component system involved in biofilm formation	[58]
*bfmR*	BfmR	QS-regulated two-component system involved in biofilm formation	[58]
*pilA*	PilA (major pilin of type IV pili)	Twitching motility and evasion of the host immune system	[59]
*ompA*	OmpA	Antibiotic- and serum-resistance, biofilm formation, host-interaction, cytotoxicity, interference with autophagy and apoptosis	[60,61,62,63,64,65,66,67]
*carO*	CarO	Resistance to carbapenems	[68,69,70]
*omp33*	Omp33 (also known as Omp33-36 kDa or Omp34)	Induction of apoptosis and modulation of autophagy	[71,72]
*occAB1*	OccAB1 (also known as OprD-like or porinD)	Uptake of antibiotics and iron, host-interaction	[70,73,74,75,76]
*ompW*	OmpW	Iron uptake and cytoxicity	[77]
Acinetobactin gene cluster	Acinetobactin	Iron chelator	[78]
Fimsbactins gene cluster	Fimsbactins A-F	Iron chelators	[78]
Baumannoferrin gene cluster	Baumannoferrin A-B	Iron chelators	[78]
*fur*	Fur	Iron metabolism transcriptional regulator	[79]
*plc1* and *plc2*	PLC	Lipolytic activity for iron acquisition	[80]
*pld1-3*	PLD	Lipolytic activity for iron acquisition	[81]
*oxyR*	OxyR	ROS response regulator	[82]
*soxR*	SoxR	Superoxide response regulator	[83]
*znuA, znuCB, znuD1* and *znuD2*	ZnuA, ZnuB, ZnuC, ZnuD1 and ZnuD2	Uptake of zinc	[84]
*zur*	Zur	Zinc metabolism transcriptional regulator	[84]
*zigA*	ZigA	Zinc metallo-chaperone	[85]
*mumT*	MumT	Uptake of manganese	[86]
*pit*	PIT system	Low affinity phosphate uptake system	[87]
*pst operon*	PstS	High affinity phosphate uptake system	[88]
*phoB* and *phoR*	PhoB and PhoR	Two-component regulatory system for phosphate uptake	[89]
*hlyB, hlyD* and *tolC*	HlyB, HlyD and TolC (T1SS)	Secretion of proteins involved in biofilm formation and adhesion to pulmonary epithelia	[12]
*gsp genes*	T2SS	Secretion of proteins from the Sec or the Tat translocons	[90]
*tra* locus	T4SS	Conjugative transfer of DNA, plasmids, and other mobile genetic elements	[91]
*AbfhaB* and *AbfhaC*	AbFhaB and C (T5bSS)	Adhesion to integrin and fibronectin	[92]
*cdiA_1_*, *cdiB_1_*, *cdiA_2_* and *cdiB_2_*	CdiA and B (T5bSS)	Killing of bacterial competitors	[93]
*ata*	Ata (T5cSS)	Adhesion to collagen I, III, IV, V and laminin	[94]
Core, accessory and regulatory genes	T6SS	Contact-dependent secretion of substrates into competitor bacterial or eukaryotic cells	[95]
None	OMVs	Long-distance delivery of multiple packaged virulence factors	[96]

## Abbreviations

The following abbreviations were used in this manuscript:

ABC, ATP binding cassette; AHLs, Acyl Homo-serine Lactones; ACB complex, *Acinetobacter calcoaceticus–Acinetobacter baumannii* complex; Ata, *Acinetobacter* trimeric autotransporter; ATG, autophagy-related proteins; Bap, biofilm-associated protein; CEACAM, carcinoembryonic antigen-related cell adhesion molecules; CDI, contact-dependent inhibition; ChoP, phosphorylcholine; CRISPR, interspaced short palindromic repeat; CU, chaperone-usher; DAMP, damage-associated molecular pattern; DC, dendritic cell; ECM, extracellular matrix; Erk1/2, extracellular signal-regulated kinase 1/2; GPI, glycosylphosphatidylinositol; Gsp, general secretory protein; IC, international clone; ICU, intensive care unit; Ig, immunoglobulin; IgCAM, immunoglobulin cell adhesion molecule superfamily; IL, interleukin; IM, inner membrane; ITIM, immunoreceptor tyrosine-based inhibitory motif; Jak/STAT, Janus kinase/signal transducers and activators of transcription; JNK, c-Jun N-terminal kinase; LAP, LC3 associated phagocytosis; LC3, microtubule-associated protein light chain 3; LC3BII, lipidated form of LC3; LOS, lipo-oligosaccharide; LPS, lipopolysaccharide; MAPK, mitogen-activated protein kinase; MATE, multidrug and toxic efflux; MCP, monocyte chemoattractant protein; MDR, multidrug-resistant; MFS, major facilitator superfamily; MIP, macrophage inflammatory protein; MLST, multilocus sequence type; NFkB, nuclear factor-kappa B; NET, neutrophil extracellular trap; NK, natural killer; NOD, nucleotide-binding oligomerization domain; NOX, nicotinamide adenine dinucleotide phosphate oxidase; NRAMP, resistance-associated macrophage protein; OC, Outer Core locus; OM, outer membrane; Omp, outer membrane protein; OMV, outer membrane vesicle; PACE, proteobacterial antimicrobial compound efflux; PAF, platelet-activating factor; PAFR, platelet-activating factor receptor; PAMP, pathogen-associated molecular patterns; PLC, phospholipase C; PLD, phospholipase D; PNAG, poly-β (1-6)-N-acetyl-glucosamine; PRRs, pattern recognition receptors; Pst, phosphate-specific transporter; PTS, phosphate transport system; QS, quorum sensing; Rab, Ras related protein; RGD, arginine-glycine-aspartate; *rms*, restriction-modification system; RND, resistance nodulation-division; ROS, reactive oxygen species; RTX, repeats in toxin; SMR, small multidrug resistance; STAT, signal transducer and activator of transcription; SVAIG, pentameric collagen binding consensus sequences; T1SS, type 1 secretion system; T2SS, type 2 secretion system; T4SS, type 4 secretion system; T6SS, type 6 secretion system; TAS, toxin-antitoxin system; Tat, twin-arginine translocon; TFEB, transcription factor EB; TLR, toll-like receptor; TNF-α, tumor necrosis factor-α; TPS, two-partner secretion; WHO, World Health Organization.
